# ENVIRONMENTAL EXPOSURE TO SELECTED NON-PERSISTENT ENDOCRINE DISRUPTING CHEMICALS AND POLYCYSTIC OVARY SYNDROME: A SYSTEMATIC REVIEW

**DOI:** 10.13075/ijomeh.1896.02551

**Published:** 2025

**Authors:** Magdalena Ozga, Joanna Jurewicz

**Affiliations:** Nofer Institute of Occupational Medicine, Department of Chemical Safety, Łódź, Poland

**Keywords:** BPA, triclosan, PCOS, parabens, phthalates, endocrine disrupting chemicals

## Abstract

Polycystic ovary syndrome (PCOS) is an endocrine disorder manifesting with symptoms such as irregular menstrual cycles, hyperandrogenism, and/or polycystic ovaries. The exact cause of PCOS remains unknown, but it is believed to result from a combination of genetic predisposition, insulin resistance, low-grade inflammation, and excessive androgen production. Various environmental factors, especially endocrine-disrupting chemicals (EDCs), in addition to genetic and hormonal ones, also may influence PCOS. This is one of the first systematic reviews dealing with the exposure to non-persistent endocrine disrupting chemicals and PCOS. The review summarizes the existing knowledge about the association of EDCs with PCOS based on significant findings on the toxicity of various non-persistent environmental EDCs and polycyclic ovarian syndrome risk. A systematic search of the literature was conducted in order to identify proper studies using PubMed, Scopus, Elsevier, and Springer databases. The results of the studies suggest that there is a positive association between bisphenol A (BPA), phthalates, octocrylene, and PCOS. The data concerning triclosan and PCOS were inconclusive. Additionally, no link between the exposures to parabens and PCOS was observed. These results of the presented studies highlight the urgent need for continued research on EDCs and their role in PCOS.

## Highlights

- Exposure to selected endocrine-disrupting chemicals (EDCs) may contribute to polycystic ovary syndrome (PCOS) development.
- Bisphenol A, phthalates, and octocrylene were associated with PCOS.
- No correlation between parabens and PCOS was found.
- There is an urgent need for further studies on the impact of EDCs and PCOS.

## INTRODUCTION

Polycystic ovary syndrome (PCOS) is a heterogeneous disease, diagnosed depending on the applied diagnostic criteria. This is a common endocrine disorder affecting 11–13% of women of reproductive age across the globe [1]. Generally, approx. 70% of cases remain undiagnosed. The syndrome was originally described in 1935 by Stein and Leventhal [2], while other gynaecologists had previously reported the enlarged ovaries with the presence of multiple small follicles in women.

The syndrome was formerly characterized by a combination of symptoms such as irregular menstrual cycles, hyperandrogenism, and/or polycystic ovaries [3]. Studies performed nowadays show that PCOS is a more complex disorder with significant implications for a woman's reproductive, metabolic, and psychological health, strongly impacting a patient's quality of life [4]. Some other manifestations include anovulatory menstrual cycles, infertility, hirsutism, obesity, and an increased risk of diabetes, hypertension, lipid abnormalities, and metabolic syndrome. In general, PCOS women suffer from menstrual irregularities, ultrasound findings of abnormal ovarian size and morphology with numerous cysts, and clinical or laboratory evidence of hyperandrogenism [5].

Several sets of criteria are used by physicians to diagnose PCOS. The Rotterdam criteria from 2003 are the most widely accepted criteria, and they demand meeting 2 out of 3 of the conditions: oligo-or anovulation, clinical and/or biochemical signs of hyperandrogenism, and/or polycystic ovaries visible on ultrasound [6,7]. The National Institute of Health (NIH) criteria from 1990 require both hyperandrogenism and chronic anovulation [6]. The Androgen Excess Society (AES) criteria established in 2006 prioritize hyperandrogenism as a necessary criterion along with ovarian dysfunction and/or polycystic ovaries, but they have never been widely adopted for PCOS diagnosis [2,8].

Diagnosing PCOS is not straightforward, as there is no single test for this complex disease. Physicians often diagnose PCOS based on a combination of a woman's symptoms, medical history, and a physical examination. Hormone level tests and pelvic examinations to evaluate the ovaries are commonly used tools [8]. Ultrasound scans can also provide insight into the appearance of the ovaries and the uterine lining thickness. Early diagnosis and treatment, combined with weight loss, may mitigate the risk of long-term consequences like type 2 diabetes and heart disease. However, diagnosing PCOS often requires extended timelines and multiple health-care appointments and should be taken seriously [1]. Receiving a diagnosis of this syndrome is associated with significant psychological distress, reduced well-being, depression, anxiety, and concerns about future health and fertility for patients [9]. Polycystic ovary syndrome is a multifactorial disease [2]. The scientists made an effort to identify the pathogenetic pathways that underlie PCOS. Although huge progress has been made, the exact etiology of PCOS is unknown, but it is likely a combination of factors, including genetics, insulin resistance, low-grade inflammation, and excess androgen production, although the actual role of androgen and the related signals in PCOS have not yet been clarified [10]. Over 241 candidate genes have been linked to various phenotypes of PCOS and identified as contributors to the disease's pathophysiology.

Polycystic ovary syndrome is influenced not only by genetic and hormonal factors but also by various environmental and nutritional factors [11]. These factors can worsen symptoms and affect the overall health of women with PCOS. Particularly endocrine-disrupting chemicals (EDCs), described widely in the recent literature, are substances that can interfere with hormonal systems, and exposure to these can affect the development and severity of PCOS and impact the pathomechanisms of PCOS [12]. Although endocrine disruption has only lately received high attention, the phenomenon has been known for a long time, almost since the identification of the first hormone in 1902. Early indications of an endocrine-disrupting activity were reported in the 1920s in the wildlife population. At present, according to the Endocrine Society, approx. 1000 chemicals worldwide are defined as EDCs based on their probable endocrine-interfering properties [13]. Endocrine-disrupting chemicals are a class of natural or man-made chemicals that can interfere with the body's hormonal system [14]. These chemicals can mimic, block, or alter the way hormones naturally work, potentially leading to a variety of health problems [15]. Humans are exposed to EDCs through ingestion, inhalation, and direct contact through the skin.

The EDCs fit into broad general categories of chemicals: heavy metals, industrial, pharmaceutical, residential, and agricultural. They are found in many everyday products, including food, drugs, plastics, personal care products (cosmetics, soaps, shampoos, and sunscreens), pesticides and herbicides, food packaging and linings, and industrial chemicals (flame retardants and solvents) [16]. Endocrine-disrupting chemicals, based on their tendency to endure in the environment and the human body, can be divided into 2 groups. Non-persistent EDCs are characterized by a shorter half-life and lower liposolubility [17]. Persistent EDCs last longer in the environment and potentially accumulate in living organisms. These substances can be present for years, if not decades, and continue to affect the endocrine system [18].

Endocrine-disrupting chemicals can disrupt a balance of variety of hormones, including estrogen, testosterone, thyroid hormones, and others [18], potentially causing a wide range of human health problems, including reproductive [19] and, developmental issues [20], metabolic disorders [21], malignancies [22], and neurological difficulties [23]. Polycyclic ovarian syndrome is one of the health effect suspected to be associated with exposure to EDCs. Although numerous human studies have been conducted on the influence of exposure to non-persistent endocrine-disrupting chemicals on PCOS [15,24,25] the results are inconsistent and there are just a few studies on specific type of non-persistent endocrine disrupting chemicals which make difficult to receive the proper conclusion. These studies highlight the need for continued study on already existing and newly appearing EDCs. Taking into account the continuous presence of EDCs in everyday products such as plastics, personal care items, and food packaging, it is critical to understand their long-term effects on human reproductive health, especially polycystic ovarian syndrome.

In present review, relationship between human exposures to certain non-persistent endocrine disrupting chemicals (bisphenols, phthalates, parabens, UV filters, and triclosan (TCS)) and polycystic ovary syndrome was evaluated. By summarizing current epidemiologic knowledge and identifying areas for further research, the review intend to provide a deep understanding of how these chemicals influence PCOS.

## METHODS

This review adhered to the PRISMA (preferred reporting items for systematic reviews and meta-analyses) guidelines. A comprehensive search of the literature was performed to identify relevant English-language studies published in 2010–2024. References within these studies were also reviewed to uncover additional pertinent research. The databases used for the search included PubMed, Scopus, Elsevier and Springer. Search terms encompassed combinations of keywords related to exposure to non-persistent chemicals and polycyclic ovarian syndrome, such as bisphenols, phthalates, parabens, UV filters (benzophenones, homomethyl salicylate (HMS), octocrylene (OC)) and TCS.

The review focused on recent human studies published in peer-reviewed English journals from 2010 onward, reflecting the past 14 years of research. This period was selected due to significant findings on the toxicity of various nonpersistent environmental EDCs, particularly concerning polycyclic ovarian syndrome risk.

A total of 94 articles were identified through the search. Each was evaluated for eligibility, and additional articles were found by manually reviewing the references of selected studies.

From the initial pool, 18 publications on exposure to nonpersistent endocrine-disrupting chemicals and polycyclic ovarian syndrome were chosen by 2 reviewers, who demonstrated high concordance (κ = 0.80). This review included original peer-reviewed studies focused on human exposure to non-persistent EDCs and polycyclic ovarian syndrome. Studies with duplicate data, those published before 2010 and those examining other endocrinological disorders (such as uterine leiomyoma, endometriosis, and recurrent miscarriages) or occupational exposures were also excluded. Additionally, animal research, *in vitro* studies, review papers, and non-English publications were not considered.

Two researchers independently identified and assessed relevant articles for inclusion, resolving discrepancies through discussion and the involvement of a third independent author when necessary. The selection process involved screening titles and abstracts, eliminating duplicates and irrelevant items, and conducting a full-text review of the remaining articles. Each full-text article was thoroughly examined to determine the study's aims, statistical methods, and results.

The following data were extracted from each study for this review: study population, type and assessment methods of exposure (including biomarkers), type of study, level of exposure to the EDCs, and study results. All included articles were summarized and discussed comprehensively.

## RESULTS

### Exposure to bisphenols (specifically BPA and its analogues) and PCOS

Bisphenols are a class of chemical compounds that are mainly used as monomers in the production of plastics and resin [26]. Bisphenol A (BPA) is the most studied compound among the bisphenol group, but due to its endocrine disrupting effect, its usage in some countries is prohibited. Its analogues, bisphenol S (BPS) and bisphenol F (BPF), were synthesized in order to overcome these issues, but the studies indicate that they also may have the same effects on human health [27].

Bisphenol A (2,2-bis-(4-hydroxylphenol) propane) is a chemical compound used in the production of polycarbonate plastics (e.g., plastic water bottles, food storage containers, beverage cans lined with epoxy resin, thermal paper receipts, dental sealants, and medical devices) [26]. It is classified as EDC because it can act as a xenoestrogen [28]. Bisphenol A can interfere with the hormonal system, mimicking or blocking hormones (mainly estrogen hormone) and disrupting their normal physiological functions [29,30].

Bisphenol A exposure has been linked to development issues during the prenatal and early childhood stages [31], cancer risk (including hormone-dependent tumours such as breast or prostate cancer), and reproductive disorders like endometriosis, infertility, and PCOS [32], although the exact role of BPA in the latter condition is still unknown. In February 2024, the European Commission proposed a new draft aimed at amending the existing regulations on food contact materials (FCMs), involving a ban on BPA and its derivatives. Since 2020, BPA has been banned from thermal paper receipts.

Bisphenol A can be detected in various human body fluids, including urine, serum, saliva, follicular fluid, breast milk, colostrum, placenta, umbilical cord, and amniotic fluid [33].

In this review, the authors examine the relationship between BPA and its analogues, and PCOS based on 12 publications [34–45] (Table 1), including 10 case-control studies [34,36–39,41–45] and 2 cross-sectional studies [35,40]. The studies were conducted in India, the United Kingdom, Poland, Iran, Egypt, USA, the Czech Republic, and China.

**Table 1.** Association of non-persistent endocrine disrupting chemicals (EDC) (bisphenols, phthalates, parabens, UV filters and triclosan) with polycystic ovary syndrome (PCOS) in women – summary of English-language studies published in 2010–2024

Study	Study population	Study design	Sample type	EDC concentration	Results
BPA and its analogues					
Kawa et al. (2018), India [34]	49 KOS women recruited from various hospitals (Endocrinology and Gynecology Outpatient Department under guidance of specialists). The diagnosis was based on Rotterdam criteria 2003 which is defined by the occurrence of 2 of the following 3 criteria: menstrual dysfunction (intermenstrual penods >35 days or <21 days), clinical and/or biochemical hyperandrogenlsm (hirsutism and/or elevated total 1ST) and the ultrasonographic presence of polycystic ovary morphology (> 12 follicles in either or both ovaries measuring 2–9 mm in diameter). Control group consisted of 39 healthy volunteers recruited from various screening camps conducted at university and various colleges with regular menstrual cycles, no evidence of hirsutism, acne and hyperandrogenemia.	case-control study	blood serum LOD – no information	AM±SD[ng/ml] PCOS26.4±14.9, controls 18.95±8.88	The PCOS women showed statistically significant increase in serum BPA levels as compared to healthy women (p = 0.0046). The BPA level was also significantly associated with the clinical, hormonal, metabolic and haematological parameters as follows: waist circumference p = 0.027, BMI p = 0.039, TST p = 0.001, blood glucose fasting p = 0.001, blood glucose −1 h p = 0.032, blood glucose −2 h p = 0.005, serum total cholesterol p = 0.011, triglycerides p = 0.011, insulin fasting p = 0.002, HOMA-IR p <0.0001, QUICKI p = 0.0009, HCT p = 0.048 and MCV p = 0.011.
Kandaraki et al. (2010), UK [35]	The PCOS group comprised 71 women who were referred to the PCOS endocrine clinic due to menstrual irregularities. The diagnosis of PCOS was based on NIH consensus criteria. Chronical anovulation was assessed by <8 cycles/year and serum progesterone levels were <3 ng/ml during the study period. Hyperandrogenemia was assessed as total TST levels above the 95th percentile of the levels detected in the group of normal menstruating women. 100 healthy women with regular periods and no hyperandrogenemia, hirsutism, or acne served as the control group, studied during follicular phase. The studied subjects were subdivided into 2 groups according to their BMI: the lean (<25 kg/m^2^) and the overweight (≥25 kg/m^2^).	cross-sectional study	blood serum LOD – no information	AM±SD [ng/ml] PCOS 1.05±0.56, controls 0.72±0.37, lean PCOS 1.13±0.63, lean controls 0.70±0.36, obese PCOS 0.96±0.46, obese controls 0.72±0.39	Serum BPA concentration was significantly higher in the PCOS group compared with controls (p < 0.0001). In lean women with PCOS BPA value was significantly higher compared with lean controls and the same difference was observed in the obese subgroups (p < 0.05).
Rutkowska et al. (2020), Poland [36]	35 PCOS women according to the ES H RA/ASRM criteria (Rotterdam criteria) and showing 2 out of the following 3 features: clinical or laboratory indices of androgen excess, chronic anovulation, the presence of polycystic ovarian morphology visible on transvaginal ultrasonography. The control group consisted of 44 women without any endocrinopathy and not taking any hormonal contraceptives. All the women were recruited at the Department and Clinic of Endocrinology, Diabetology and Isotope Therapy, Medical University of Wrocław, Poland.	case-control study	blood sample LOD = 0.0093 ng/ml, LOQ = 0.028 g/ml	AM±SD [ng/ml] PCOS 17.47±8.28, controls 12.97±8.19	The group with PCOS had higher serum BPA concentration than control group (p = 0.06). After dividing the participants into those with and without detectable serum BPA, no differences in the clinical and hormonal characteristics were found between these subgroups. Positive correlations between serum BPA level and TST (p = 0.05), 17β-estradiol (p = 0.029), LH/FSH ratio (p = 0.03), FAI (p = 0.007), and androstenedione (p = 0.02) were observed in PCOS patients.
Prabhu et al. (2023), India [37]	120 clinically diagnosed PCOS patients and 119 healthy control participants were enrolled for the study at the Department of Obstetrics and Gynecology, Dr. TMA Pai Hospital, Udupi, India. Patients who come to the hospital for a check-up. Women aged 18–45 years diagnosed with PCOS according to Rotterdam diagnostic criteria (2003) were included in the study.	case-control study	urine LOD – no information	creatinine-adjusted AM±SD [μg/g creatinine] PCOS 4.1±1.3, controls 3.3±1.2	The significantly elevated levels of BPA in women with PCOS than those of healthy control women was observed (p < 0.0001).
Patel et al. (2024), India [38]	130 women aged 17–40 years were recruited for the study during January 2022 – July 2023. This recruitment took place at the Dr. Nagori Institute, Ahmedabad, India. The diagnosis of PCOS in 80 of these women was based on the ESHRE/ASRM's Rotterdam Criteria, requiring the manifestation of 2 out of 3 specific features. The control group comprised 50 women, chosen from the initial pool of recruits based on their lack of any endocrinopathy and absence of hormonal contraceptive use.	case-control study	blood serum LOD = 0.01 ng/ml, LOQ = 0.04 ng/ml	AM±SD [ng/ml] PCOS 102.15±0.1, controls 61.35±50.13	The study showed that BPA levels were significantly higher in the PCOS group than in the control group (p < 0.0001).
Rashidi et al. (2017), Iran [39]	All cases were selected from women who were diagnosed with PCOS at Gynecology and infertility centre. The control group was selected from women who had clinical file in the centre due to the previous problem and came for routine check-up and Pap smear. After confirmation the PCOS by transvaginal ultrasound they entered to the study (51 women). The control group (51 women) had not any symptoms of current or previous PCOS in the clinical and sonographic examination.	case-control study	urine BPA: LOD = 0.33 μg/l	AM±SD [ng/ml] PCOS 3.34±2.63, controls 1.43±1.57	Comparison of BPA level between 2 groups shows significantly higher level in PCOS group compared with control group (p < 0.001). Using logistic regression analysis, BPA as the main dependent variable, was significantly associated with PCOS OR = 1.53 (95% Cl: 1.14–2.05, p = 0.004).
Elkafrawy et al. (2018), Egypt [40]	This study was conducted at Al Zahraa University Hospital, Cairo, Egypt, January 2017 – July 2018, on 160 infertile women, 80 of them were PCOS and the other 80 women were non-PCOS. Patients with infertility due to male factor, due to ovarian failure, with history of previous contraception or hormonal treatment for induction before the research was excluded. PCOS cases were selected according to Rotterdam criteria.	cross sectional study	urine LOD – no information	Me (IQR) range [ng/ml] PCOS 24.49 (12.97–28.66) 2.18–34.95, controls 5.63 (4.2–6.85) 1.85–30.45	Urinary BPA was increased in infertile women, PCOS and non PCOS groups. The association between BPA exposure and lower of antral follicles count (p = 0.031) and AMH level (p < 0.001) in infertile was observed.
Vagi et al. (2014), USA, California [41]	52 PCOS case patients diagnosed using the NIH 1990 definition and 50 controls were recruited in 2007–2008 from an urban academic medical centre in Los AngeIes, USA.	case-control study	urine LOD = 0.4 mg/l	creatinine adjusted GM concentration [μg/g creatinine] PCOS 1.6, control s 1.9	PCOS case patients and controls had similar geometric mean of urinary BPA concentration.
Šimkova et al. (2020), Czech Republic [42]	The study consist of 39 patients: 19 women with PCOS divided into subgroups according to BMI – 9 normal-weight (BMI 21.4±3.2 kg/r∩2) and 10 obese (BMI 35±2.7 kg/m^2^), 20 healthy controls. All patients met NIH 1990 and ESHRE criteria. Normal-weight PCOS women were 28.9±7.4 years old, obese PCOS women 29.5±5.8 and healthy controls 29.9±6.4 years old.	case-control study	blood plasma LOD – no information	Me (Q_1_–Q_3_) [nmol/l] – BPA: normal-weight PCOS 0.282 (0.129–0.356), obese PCOS 0.129 (0.129–0.192), controls 0.129 (0.065–0.211) – BPS: normal-weight PCOS 0 (0, 0.155), obese PCOS 0 (0–0.155), controls 0 (0–0.391)	The exposure to BPA was significantly higher in normal-weight PCOS women than in healthy controls (p = 0.042).
Gu et aL (2019), China [43]	123 infertile women at 18–45 years of age who came to Women's Hospital, Zhejiang University School of Medicine, China, March 2014 – August 2015: 40 women diagnosed with PCOS according to the Rotterdam criteria and 83 healthy women with regular menstrual cycles; no endocrine disorders were recruited as controls.	case-control study	urine LOD range = 2 fg/ml – 0.5 pg/ml	creatinine adjusted Me [μg/g creatinine] PCOS <LOD controls <LOD	There were no significant relationship between PCOS and urinary BPA.
Jurewicz et al. (2021), Poland [44]	The study consists of 199 women with PCOS and 158 control women. All the study subjects were recruited in the period of January 3 – December 21, 2017 at the medical practice. Inclusion criteria were age 18–40 years and the diagnosis of PCOS according to the AES&PCOS criteria where apart from clinical or biochemical signs of hyperandrogenism, ovulatory disorder or/and PCOM. The control group consisted of healthy women, who were referred to the same practice in order to exclude some of the common endocrine disorders (i.e.z hypothyroidism or hyperprolactinaemia).	case-control study	blood serum – BPA: LOD = 0.009 ng/ml; LOQ = 0.028 ng/ml – BPS: LOD = 0.022 ng/mlz LOQ = 0.067 ng/ml – BPF: LOD = 0.012 ng/ml, LOQ = 0.037 ng/ml	GM (95% Cl) [ng/ml] – BPA: PCOS 0.46 (0.42–0.47), controls 0.33 (0.30–0.37) – BPS: PCOS 0.14z controls 0.08 – BPF: PCOS 0.10 (0.09–0.14), controls: 0.09 (0.07–0.11)	Serum BPA and BPF concentrations did not differ between the studied groups. There was a negative correlation between serum BPA and HOMA-IR (p = 0.001) and TST (p = 0.006) in women with PCOS. No correlations were found between the serum BPS and other metabolic parameters such as serum lipids, glucose, insulin, DHEA-S, androstenedione and FAI
Majewska et al. (2024), Poland [45]	The study included 135 women diagnosed with PCOS and 104 healthy controls aged 17–45 years. The diagnosis of PCOS was made according to the AES&PCOS.	case-control study	blood serum – BPE: LOD = 0.011 ng/ml, LOQ = 0.032 ng/ml – BPC: LOD = 0.021 ng/ml, LOQ = 0.061 ng/ml – BPG: LOD = 0.008 ng/ml, LOQ = 0.024 ng/ml – BPM: LOD = 0.018 ng/ml, LOQ = 0.054 ng/ml – BPP: LOD = 0.019 ng/ml, LOQ = 0.056 ng/ml – BPZ: LOD = 0.17 ng/ml, LOQ = 0.051 ng/ml – BPFL: LOD = 0.014 ng/ml, LOQ = 0.041 ng/ml – BPBP: LOD = 0.029 ng/ml, LOQ = 0.12 ng/ml	GM (95% Cl) [ng/ml] – BPE: PCOS 0.037 (0.013–0.185), controls 0.039 (0.011–0.152) – BPC: PCOS 0.029 (0.009–0.890), controls 0.033 (0.018–0.832) – BPG: PCOS 0.052 (0.016–0.278), controls 0.054 (0.021–0.268) – BPM: PCOS 0.067 (0.013, 0.115), controls 0.058 (0.033–0.123) – BPP: PCOS 0.049 (0.009–0.129), controls 0.047 (0.017–0.146) – BPZ: PCOS 0.084 (0.027–0.157), controls 0.092 (0.025–0.193) – BPFL: PCOS 0.011 (0.008–0.127), controls 0.010 (0.006–0.129) – BPBP: PCOS 0.031 (0.009–0.754), controls 0.030 (0.011–0.614)	Serum BPE, BPC, BPG, BPM, BPP, BPZ, BPFL and BPBP concentrations did not differ significantly between PCOS and control subjects. Women whose serum BPM and BPP concentrations were in the highest tertile were more likely to be PCOS diagnosed with PCOS (adjusted OR = 0.43 [95% Cl 0.20–0.89], p < 0.001 and 0.56 [95% Cl: 0.27–0.96], p = 0.049, respectively).
Phthalates					
Milankov et al. (2023), Serbia [56]	60 female in reproductive age with already confirmed PCOS according to the Rotterdam criteria based on the presence of at least 2 out of 3 following criteria: chronic anovulation, clinical or biochemical signs of hyperandrogenism and ultrasound-confirmed polycystic ovaries. All participants were divided into 2 subgroups based on the presence of analysed phthalates in the urine sample. The subjects who had at least 1 phthalate above LOD in urine sample were phthalates +, while subjects with no phthalate metabolite in urine samples were labelled as phthalates –.	cross-sectional study	urine each phthalate monoester: LOD = 0.25 ng/ml, LOQ = 0.5 ng/ml	creatinine-adjusted AM±SD [μg/g creatinine] of the sum (Σ) ofall phthalates: 9.57 ±11.66 (range 0.51–54.23) – MMP: 8.13±10.63 – MEP: 12.52±6.51 – MPP: 2.06±0.96 – MBP: 3.10±0.00 – MiAP:– – MnAP:– – MCHP: 10.70±0.00 – MBzP:– – MOP: 3.44±6.83 – MEHP: 6.91±6.83	Total cholesterol level was statistically significantly higher among PCOS women with detected phthalate metabolites compared to those without phthalates (p≤ 0.05). A statistically significant positive correlation was found between phthalates metabolites sum and anthropometric parameters: BMI p = 0.029, waist circumference p = 0.011, waist-to-high ratio p = 0.004. Leptin serum levels were positively correlated with the urine sum of phthalates levels (p = 0.005). There was a statistically significant correlation between the sum of phthalates determined in the urine samples and derived parameters LAP (p = 0.003) and VAI (p = 0.003). The sum of phthalates metabolites was also positively associated with glucose metabolism parameters such as fasting plasma glucose (p = 0.018) and HOMA index values (p = 0.027). Total cholesterol (p = 0.017), triglycerides (p = 0.009) and LDL cholesterol (p = 0.023) were related to the sum of total phthalate metabolites. Testosterone serum levels were positively associated with sum of phthalate metabolites detected in the urine sample with borderline significance (p = 0.058).
Vagi et al. (2014), USA, California [41]	52 PCOS case patients diagnosed using the NIH 1990 definition and 50 controls were recruited in 2007–2008 from an urban academic medical centre in Los AngeIeszUSA.	case-control study	urine phthalates metabolites LOD range = 0.2–1.2 mg/l	Creatinine-adjusted GM [μg/g creatinine] – MBzP: PCOS 7.5, controls 11.7 – MBP: PCOS 17.7, controls 23.2 – MEP: PCOS 181.1, controls 195.8 – MCNP: PCOS 3.6, controls 2.6 – MCOP: PCOS 7.8, controls 8.4 – MCPP: PCOS 2.8, controls 3.4 – MECPP: PCOS 47.9, controls 40.4 – MEHHP: PCOS 27.3, controls 26.4 – MEHP: PCOS 3.2, controls 3.5 – MEOHP: PCOS 16.2, controls 16.3 – MiBP: PCOS 7.0, controls 8.2	PCOS case-patients had lower urine concentrations of mBP (p = 0.02) and mBzP (p < 0.05). Lower concentrations of MBzP, mBP, MEHP and MEP were associated with an increased likelihood of PCOS.
Akin et al. (2020), Turkey [57]	The study included adolescent girls who were presented to the Paediatric Endocrinology Outpatient Clinic because of irregular menstrual bleeding and/or hirsutism between January 2011 – August 2012. An age-matched cohort who had regular menstruation and did not have hirsutism served as the control group. The modified Rotterdam criteria were used for the diagnosis of PCOS and the adolescents who had BMI ≥95th percentile according to age and sex were defined as obese. Subjects required any 2 of the following 3 criteria to be present to be diagnosed with PCOS: oligo/anovulation, clinical and/or biochemical evidence of hyperandrogenism and PCOM on ultrasound, with other endocrinopathies excluded. In total 63 PCOS and 61 control adolescent girls were included. Participants were further subdivided into lean and obese subgroups according to BMI.	cross-sectional study	blood serum the detection limits: – DEHP: 0.05 ppm – MEHP: 1 ppm	AM (95%CI) [mg/ml] – DEHP: PCOS 2.62 (2.50–2.75), controls 2.71 (2.52–2.90) GM (95% Cl) [mg/ml] – MEHP: PCOS 0.23 (0.19–0.29), controls 0.36 (0.18–0.54)	Serum DEHP and MEHP concentration were not significantly different between PCOS and control groups. Correlation analysis, adjusted for BMI, showed that both phthalates significantly correlated with insulin resistance indices and serum triglycerides in adolescents with PCOS (p < 0.05).
Xu et al. (2011), China [58]	18 patients with PCOS (PCOS group) and 16 patients without PCOS or laparoscopy – proven endometriosis but with infertility associated with tubal defects or pelvic adhesion (control group). The diagnosis of PCOS was based on the criteria from 2003 Rotterdam conference: oligo- or anovulation, clinical signs of hyperandrogenism and/or hyperandrogenism, polycystic ovaries: the number of follicles with a diameter of 2–9 mm in 1 or both ovaries ≥12, and or ovarian volume ≥10 ml. Inclusion of 2 of 3 above criteria and exclusion of other causes of hyperandrogenism: congenital adrenocortical hyperplasia, Caushing syndrome, androgen-secreting tumors, as well as other diseases leading to ovulation disorders such as hyperprolactinemia, and abnormal thyroid function. All patients in this study did not take hormones in latest 3 months, and signed the consent before study.	case-control study	blood serum the lower detection limits: – DEP: Y = 49713.8X+3190.3, r = 0.9998 – DBP: Y = 39911.2X+691.7, r = 0.9999 – DEHP: Y = 30402.7 X+1155.4, r = 0.9999	AM concentration±SD [mg/ml] – DEP: PCOS 0.45±0.24, controls 0.26±0.10 – DBP: PCOS 0.53±0.15, controls 0.41 ±0.14 – DEHP: PCOS 0.41±0.12, controls 0.40±0.11	Results showed PCOS patients had significantly higher levels of DEP and DBP than those in control group (p < 0.05). Meanwhile, the levels of DBP, DEP, and DEHP declined in sequence in PCOS women. But no statistically significant difference in DEHP level was noted between these 2 groups (p = 0.853); in the PCOS patients, there was no significant correlation between the serum levels of 3 phthalate esters (DBP, DEP, and DEHP) and estradiol, TST, FSH, LH, prolactin, BMI, and age.
Jin et al. (2019), China [59]	56 infertile women with PCOS and 51 infertile women with tubal blockage (controls). The women with PCOS were diagnosed according to the revised 2003 Rotterdam criteria, indicating PCOS to be present if at least 2 of the following 3 criteria are met: oligo- or anovulation, clinical and/or biochemical signs of hyperandrogenism, and polycystic ovaries viewed on an ultrasound. Inclusion criteria were 20–45 years of the age, BMI ≤35 kg/m^2^, and undergoing IVF. The controI subjects had regular menstrual cycles and normal sex hormone levels. No structural abnormalities of the uterus and ovaries were found by vaginal ultrasound or laparoscopy in any of the women.	case-control pilot study	follicular fluid LOD – no information	GM (95% Cl) [ng/ml] PCOS 1.68 (1.27–2.13), controls 1.21 (1.06–1.38)	Concentrations of DEHP in follicular fluid were significantly higher in women with PCOS than in controls (p < 0.05). The clinical pregnancy rate was significantly lower in women with PCOS with high levels of DEHP than in controls.
Parabens					
Šimkova et al. (2020), Czech Republic [42]	The study consist of 39 patients: 19 women with PCOS divided into subgroups according to BMI: 9 normal-weight (BMI M±SD 21.4±3.2 kg/m^2^) and 10 obese (BMI 35±2.7 kg/m^2^), 20 healthy controls. All patients met NIH 1990 and ESHRE criteria. Normal-weight PCOS women were M±SD 28.9±7.4 years old, obese PCOS women 29.5±5.8 years old, and healthy controls 29.9±6.4 years old.	case-control study	blood plasma LOD – no information	Me with lower and upper quartiles: ∑ of parabens [nmol/ml] controls – not detected, normal-weight PCOS 0.488, obese PCOS – not detected	No association of parabens and PCOS in women.
UV filters (BP-3, HMSz OC)					
Gu et al. (2019), China [43]	The study utilized resources from the National Basic Research Program of China (Program 973), a project comprising 2178 women from Shandong, Zhejiang province and Shanghai, China, focusing on the impact of environmental endocrine disruptors on female reproductive function, in which 397 were diagnosed with PCOS. Due the territory distance and quantity of urine sample provided by eligible women, this retrospective case-control study included 123 women with no history of pregnancy, 20–41 years of age. Those who had other endocrine diseases or whose mother had endocrine or metabolic disorder were excluded. The study consist of 40 women who were diagnosed with PCOS according to the Rotterdam criteria, which required the presence of at least 2 of the following clinical or laboratory abnormalities: oligo-ovulation or anovulation, elevated levels of circulating androgens or their clinical manifestation and polycystic ovaries, as defined by ultrasonography. 83 healthy women with regular menstrual cycles and no endocrine disorders were recruited as control subjects.	case-control study	urine LOD range = 2 fg/ml – 0.5 pg/ml	creatinine adjusted Me [μg/g creatinine] – BP-3: PCOS 0.59, controls 14.16 – HMS: PCOS <LOD, controls <LOD – 0C: PCOS 1.58, controls 2.51	No significant relationship between PCOS and urinary BP-3 (p=0.237). No association between HMS concentration and PCOS (p = 0.830). Urinary OC concentration and PCOS were significantly associated in women with BMI ≥24 kg/m^2^ (adjusted OR = 1.512, 95% Cl: 1.043–2.191). There is a positive association between OC and PCOS risk in obese and overweight women.
Triclosan					
Ye et al. (2018), China [86]	296 infertile women at 18–45 years of age who came to the Women's Hospital, Zhejing University SchooI of Medicine, China, for infertility, March 2014 – August 2015. Infertility cases due to genital tract malformation with or without surgery were excluded from the study. PCOS was diagnosed according to the revised 2003 Rotterdam criteria. The study was limited to 84 infertile PCOS cases and 212 infertile controls.	cross-sectional study	urine L0D = 0.1 ng/ml	creatinine adjusted Me (IQR) [μg/g creatinine] – triclosan: PCOS 1.49 (0.68–3.80), controls 1.06 (0.52–3.02)	Infertile women with PCOS had a significantly higher triclosan level than infertile women without PCOS (p = 0.0407)
Gu et al. (2019), China [43]	123 infertile women at 18–45 years of age who came to Women's Hospital, Zhejiang University School of Medicine, China, March 2014 – August 2015: 40 women diagnosed with PCOS according to the Rotterdam criteria and 83 healthy women with regular menstrual cycles; no endocrine disorders were recruited as controls.	case-control study	urine LOD range = 2 fg/ml – 0.5 pg/ml	creatinine adjusted Me [μg/g creatinine] – triclosan: PCOS <LOD, controls <LOD	No association between urinary triclosan and PCOS (p = 0.651).

AM – arithmetic mean; GM – geometric mean; IQR – interquartile range; LOD – limit of detection; LOQ – limit of quantification.

AES&PCOS – Androgen Excess and PCOS Society; ASRM – The American Society for Reproductive Medicine; ESHRE – European Society of Human Reproduction and Embryology; NIH – The National Institute of Health.

AMH – Anti-Müllerian hormone; FSH – follicle stimulating hormone; HCT – hematocrit; LAP – lipid accumulation product; LDL – low density lipoprotein; LH – luteinizing hormone; TST – testosterone.

BPA, C, E, G, M, P, Z, FL, BP – bisphenol A, C, E, G, M, P, Z, FL, BP; BP-3 – benzophenone-3; DBP – dibutyl phthalate; DEHP – diisoctyl phthalate; DEP – diethyl phthalate; DHEA-S – dehydroepiandrosterone sulphate;

HMS – homomethyl salicylate; MEHP – mono-2-(ethylhexyl) phthalate; MBP – mono-n-butyl phthalate; MBzP – mono-benzyl phthalate; MCHP – mono-cyclohexyl phthalate; MCNP – mono(7-carboxy-2methyloctyl)

phthalate; MCPP – mono(3-carboxypropyl)phthalate; MEHHP – mono(2-ethylhexyl)phthalate; MEOHP – mono(2-ethyl-5-oxohexyl)phthalate; MEP – monoethyl phthalate; MiAP – mono-iso-allyl phthalate;

MiBP – mono-isobenzyl phthalate; MMP – monomethyl phthalate; MnAP – mono-n-allyl phthalate; MOP – mono-n-octyl phthalate; MPP – mono-n-pentyl phthalate; OC – octocrylene.

FAI – free androgen index; HOMA – homeostatic model assessment; HOMA-IR – homeostatic model assessment – insulin resistance; IVF – in vitro fertilization; MCV – mean corpuscular volume;

PCOM – polycystic ovary morphology; QUICKI – quantitative insulin sensitivity check index; VAI – visceral adiposity index.

The concentrations of bisphenols were primarily determined in urine samples (5 publications [37,39–41,43]), serum (6 publications [34–36,38,44,45]) and plasma samples (1 publication [42]).

The PCOS diagnosis was based on many different criteria, including the most commonly used Rotterdam criteria (8 studies [34,36–40,42–43], NIH 1990 criteria (3 studies [35,41,42]), and AES (2 studies [44–45]).

A significant increase in the serum BPA level of PCOS women was found in comparison to healthy controls in 4 studies [34–36,38], vs. 2 publications in which the serum concentration of BPA and its analogues did not significantly differ between the studied groups [44,45]. Regarding blood plasma, the exposure to BPA was significantly higher in normal-weight PCOS women than in healthy controls (p = 0.042) [42].

A comparison of urinary levels of BPA showed a significantly elevated concentration in women with PCOS compared to healthy controls in 2 study populations [37,39]. Furthermore, urinary BPA concentration was increased in infertile women, PCOS, and non-PCOS groups based on Elkafraway et al. [39]. There were similar concentrations of urinary BPA for PCOS patients and healthy controls in 2 studies [41,43], indicating no link between PCOS and exposure to BPA. The majority of studies indicate an association between BPA exposure and PCOS both when the exposure assessment was based on blood (5 publications) or urinary (3 publications) samples.

### Exposure to phthalates and PCOS

Phthalates, or phthalate esters are esters of phthalic acid. They form a large, diverse group of chemicals used as plasticizers to improve the flexibility, transparency, and durability of plastics [46] in production toys and FCM. Phthalates are also used as solvents in personal care products, such as perfumes, lotions, and cosmetics (hair spray, nail polish) [47], as well as medical devices (tubing for intravenous administration, blood bags) [48].

Many countries have implemented regulations to limit the use of phthalates, although they are neither unified nor seem adequate, as many studies suggest that more sensitive populations like children or pregnant women are exposed to phthalates that should be restricted [49]. Especially, 7 of the most used phthalates – di(2-ethylhexyl) phthalate (DEHP), butylbenzyl phthalate (BBzP), dibutyl phthalate (DBP), diisobutyl phthalate (DIBP), diisononyl phthalate (DINP), diisodecyl phthalate (DIDP), di-n-octyl phthalate (DNOP) are severely restricted in the FCM, and mostly banned as cosmetic components or allow to limited concentration (0.1% by weight) in plastic toys in EU and USA [49].

Phthalates can impair the endocrine system by mimicking hormones like oestrogen, altering hormone balance, and signalling [50]. Hormonal imbalances can impair menstrual cycle regularity and fertility. Prolonged exposure to phthalates has been linked to a variety of health problems, including reproductive and developmental issues. Phthalate exposure has been associated with decreased sperm quality, testicular dysgenesis syndrome, and genital abnormalities. Exposure during pregnancy may affect foetal development and raise the risk of developmental problems [51]. Phthalate exposure in early childhood has been linked to neurodevelopmental and behavioural disorders [52]. Phthalates can contribute to respiratory issues and allergic reactions, particularly in sensitive individuals [53,54].

The metabolism of phthalate in human body is complex. The initial step involves enzymatic hydrolysis of the parent diester into its corresponding monoester. For example, DEHP is hydrolysed to mono(2-ethylhexyl) phthalate (MEHP). This hydrolysis is facilitated by esterase in the gastrointestinal tract or liver. The monoester metabolites often exhibit greater biological activity and toxicity than the parent compounds. For some high-molecular-weight phthalates (DEHP), monoester metabolites like MEHP undergo further oxidative metabolism. For example MEHP can be oxidized to mono(2-ethyl-5-hydroxy-hexyl) phthalate (MEHHP) and mono(2-ethyl-5-oxohexyl) phthalate (MEOHP). These oxidative metabolites are more water-soluble and easier to excrete [55].

In this review, the authors investigate the relationship between phthalates and PCOS based on studies conducted in Serbia, the United States, China, and Turkey (Table 1), including 2 cross-sectional studies [56,57] and 3 case-control studies [41,58,59]. Polycystic ovary syndrome was diagnosed using a variety of criteria, the most commonly used being the Rotterdam criteria (4 studies [56–59], and the NIH criteria (1 study [41]).

Phthalate concentrations were measured in urine samples (2 publications [41,56]), serum (2 publications [57,59]) and follicular fluid (1 publication [58]).

According to a study published by Milankov et al. [56] PCOS women with detectable urine phthalate metabolites exhibited significantly higher total cholesterol levels (p = 0.03) compared to those in which urine samples phthalate metabolites were below limit of detection.

Vagi et al. [41] discovered that PCOS patients had lower urine concentrations of mono-n-butyl phthalate (MBP) and monobenzyl phthalate (MBzP). Lower levels of MBzP, MBP, MEHP, and monoethyl phthalate (MEP) were related to higher risk of PCOS [41].

Xu et al. [58] found that PCOS patients had significantly higher levels of DEP and DBP in serum samples compared to the control group (p < 0.05). Meanwhile, the levels of DBP, DEP, and DEHP decreased sequentially in PCOS women. However, no statistically significant difference in DEHP level was seen between these 2 groups. In the PCOS patients, there was no significant correlation between the serum levels of 3 phthalate esters (DBP, DEP, and DEHP) and estradiol (E2), testosterone, follicle stimulating hormone, luteinizing hormone, prolactin, body mass index, and age [58].

On the other hand serum DEHP and MEHP concentrations did not differ significantly between the PCOS and control groups in the study performed by Akin et al. [57]. Adjusted for BMI correlation analysis revealed a strong correlation between both phthalates with insulin resistance indices and blood triglycerides in adolescents with PCOS (p < 0.05).

Jin et al. [59] found that concentrations of DEHP in follicular fluid were significantly higher in women with PCOS than in controls (p < 0.05). The clinical pregnancy rate was significantly lower in women with PCOS with high levels of DEHP than in controls.

In conclusion, most of the studies concerning the exposure to phthalates showed a positive correlation between at least one of the phthalate metabolite concentrations and the incidence risk of PCOS.

### Exposure to parabens and PCOS

The family of parabens contains both naturally occurring (found in green tea or blueberries) [60,61] and synthetic parabens. Parabens are esters of para-hydroxybenzoic acid and have become one of the most frequently used preservatives in cosmetics, personal care items (lotions, shampoos, makeup, deodorants) [62], pharmaceuticals (ointments, topical medications) [63], and some food products (beverages, processed foods) [64]. They inhibit the growth of microorganisms such as bacteria, yeast, and mould, extending the shelf life and stability of the aforementioned products [65].

The most known parabens are methylparaben (MP), propylparaben (PP), ethylparaben (EP), butylparaben (BP), and benzylparaben (benzylP). Since 2009, isopropylparaben, isobutylparaben, phenylparaben, benzylP, pentylparaben are banned in cosmetic products in EU and, the maximum authorized concentrations are equal to 0.4% for single ester and 0.8% for mixtures of esters in relation to the use of allowed parabens as preservatives in cosmetic products [66].

Parabens can mimic estrogen and may interfere with hormone function, but their estrogenic activity in humans is much lower than that of natural estrogen [67].

The most potent estrogenic paraben identified was BP, but it was still 10 000 times less potent than E2 (the most potent natural estrogen) [66]. The hormonal activity strength depends on the length of the side chain of parabens.

Parabens are easily absorbed by the human body, and they have been detected in human tissues and fluids, raising concerns about their potential ability to affect the endocrine system [67].

Studies have suggested possible links between paraben exposure and breast cancer by increasing the growth of breast cells [68] and, decreasing cell death, increasing metastasis and blocking breast chemotherapy agents (e.g., tamoxifen) [68], although conclusive evidence is lacking [67]. Some individuals may develop allergic reactions or skin irritation from paraben exposure [69]. Parabens can enter the environment through wastewater and have been detected in aquatic ecosystems, posing potential risks to wildlife [69].

In the review, the authors found just 1 case-control study from the Czech Republic that investigated the potential relationship of parabens in plasma with PCOS [42] (Table 1). Šimková et al. [42] in the study performed among 2 groups of women: 19 PCOS women chosen based on NIH 1990 and Rotterdam criteria and 20 healthy controls demonstrated that parabens exposure is not associated with PCOS in women.

### Exposure to UV filters and PCOS

Ultraviolet (UV) filters also known as sunscreens or sun blocks, are an important means of protection against sunburns, depigmentation, photo aging, and photo carcinogenesis. Beyond their role as sunscreens, UV filters are versatile compounds with a broad range of applications providing critical protection in cosmetics, textiles, plastic packaging, and industrial materials. Their role in enhancing the durability and safety of products underscores their importance across diverse sectors [70].

They are chemical components used in skincare and cosmetic products to protect the skin from harmful UV radiation. Depending on formulation, they can protect against UVB radiation, both UVB and UVA radiation (broad-spectrum sunscreens) and UVB, UVA and visible light (tinted broad-spectrum sunscreens) [71]. Depending on their structure they can be divided into organic, also called chemical filters (carbon-containing) and inorganic, physical filters (carbon-free) [72]. Organic UV filters (benzophenones, cinnamates, and salicylates) absorb UV radiation and convert it into less harmful heat energy. Inorganic UV filters (titanium dioxide (TiO_2_) and zinc oxide (ZnO)) reflect or scatter UV radiation [73].

Some organic UV filters have been shown to exhibit estrogenic activity and may potentially disrupt hormone function, as revealed in animal research [74,75]. Their safety is monitored by regulatory agencies, and research into their impact on human health is ongoing [71]. Recently, benzophenone-3 (BP-3) and OC, both expected new restrictions from the EU concerning the allowed maximal concentration of the substances as UV filters and body products. In this review, the authors have found only 1 case-control study from China that explored the potential association between UV filters, including BP-3, HMS, OC, and PCOS in the urine samples of women (Table 1). According to Gu et al. [43], there is no significant relationship between PCOS and urine BP-3, nor is there an association between HMS concentration and PCOS. Urinary OC concentration and PCOS were substantially linked in women with BMI ≥24 (adjusted OR = 1.512, 95% Cl: 1.043–2.191). There was a positive association between OC and PCOS risk in obese and overweight women.

### Exposure to TCS and PCOS

A phenyl ether, is a biocide with antimicrobial and preservative properties that is used in personal care products (antibacterial soaps and toothpaste), cosmetics (deodorants and antiperspirants), and household items (kitchen utensils and cutting boards) due to its ability to inhibit the growth of bacteria, fungi, and mildew [76].

Prolonged usage of TCS may contribute to the formation of antibiotic-resistant bacteria, reducing the effectiveness of antibiotics in treating infections [76]. For instance, TCS-resistant strains of microorganisms such as *Escherichia coli* and *Salmonella* have been identified [77,78].

Triclosan has been shown in animal experiments to interfere with hormone regulation, which may potentially affect hormone function in humans. Numerous studies have revealed that TCS activates estrogen receptor, increasing estrogen secretion and disrupting endocrine homeostasis. Triclos-an-induced decreased testosterone levels may contribute to reduced spermatogenesis and negatively affect sperm quality [79]. Moreover, TCS decreased thyroid hormone concentrations [80], and another study showed that TCS enhanced the expression of androgen and oestrogen-sensitive genes [81]. According to the EU regulations, TCS may be used ≤0.3% in toothpaste, hand soap, bath and shower products, deodorants, face powder and cover sticks as well as nail care products and ≤0.2% in mouth wash [82].

Triclosan, as it is lipophilic, can accumulate in fatty tissues. Research studies have found concentrations of TCS in 3 out of 5 human milk samples [83]. Triclosan has also been found in the umbilical cord blood of infants, raising concerns about the potential impact of exposure to TCS on foetal abnormalities [84]. Triclosan has been found in water, soil, and aquatic organisms. It can accumulate in aquatic species, posing risks to ecosystems and wildlife [85].

This review included only 2 studies, 1 cross-sectional study [86] and 1 case-control study [43] from China on the relationship between TCS and PCOS (Table 1). Ye et al. [86] discovered that infertile women with PCOS had significantly higher TCS urine levels than infertile women without PCOS (p = 0.0407). Gu et al. [43] found no association between urinary TCS and PCOS. All TCS concentration data in urine samples of PCOS and control women were below the limit of detection.

## CONCLUSIONS

Several human studies have already shown links between non-persistent EDCs exposure and various health issues, including PCOS. However, the complexity of how these chemicals interact with the endocrine system and contribute to conditions like PCOS necessitates further investigation. Research has revealed that non-persistent EDCs can mimic or block natural hormones, leading to disruptions in hormonal balance that may worsen PCOS symptoms. Yet, the degree to which different non-persistent EDCs affect individuals can vary based on genetic susceptibility, the level and duration of exposure, and other environmental factors.

In the present review, the authors took into consideration the association of the following non-persistent EDCs: bisphenols, phthalates, parabens, UV filters, and with PCOS (Table 1). Twelve publications reviewed the influence of bisphenols on PCOS. Eight studies reviews indicated the association between BPA and its analogues with PCOS, while 4 showed no association (Table 2).

**Table 2.** Exposure to non-persistent endocrine disrupting chemicals (EDC) (bisphenols, phthalates, parabens, UV filters and triclosan) and polycystic ovary syndrome (PCOS) in women – summary English-language studies published in 2010–2024

Chemical compound	PCOS	
	statistically significant effect	no statistically significant effect
Bisphenols		
BPA	Kawa et ai. (2018) [34], Kandaraki et ai. (2010) [35], Rutkowska et al. (2020) [36], Prabhu et al. (2023) [37], Patel et al. (2024) [38], Rashidi et al. (2017) [39], Elkafrawy et al.(2018) [40], Šimkova et al. (2020) [42]	Vagi et al. (2014) [41], Gu et al. (2019) [43], Jurewicz et al. (2021) [44]
BPF		Jurewicz et al. (2021) [44]
BPS		Jurewicz et al. (2021) [44]
BPE, BPC, BPG, BPM, BPP, BPZ, BPFL, BPBP		Majewska et al. (2024) [45]
Phthalates		
MMP	Milankov et al. (2023) [56]	
MEP	Milankov et al. (2023) [56], Vagi et al. (2014) [41]	
MPP	Milankov et al. (2023) [56]	
MBP	Milankov et al. (2023) [56], Vagi et al. (2014) [41]	
MCHP	Milankov et al. (2023) [56]	
MOP	Milankov et al. (2023) [56]	
MEHP	Milankov et al. (2023) [56], Vagi et al. (2014) [41]	Akin et al. (2020) [57]
MBzP	Vagi et al. (2014) [41]	
DBP	Xu et al. (2011) [58]	
DEP	Xu et al. (2011) [58]	
DEHP	Jin et al. (2019) [59]	Xu et al. (2011) [58], Akin et al. (2020) [57]
Parabens (MP, EP, PR BR benzyIP)		Šimkova et al. (2020) [42]
UV fiIters		
BP-3		Gu et al. (2019) [43]
HMS		Gu et al. (2019) [43]
OC	Gu et al. (2019) [43]	
Tridosan	Ye et al. (2018) [86]	Gu et al. (2019) [43]

BenzylP – benzylparaben; BP – butylparaben; MP – methylparaben; EP – ethylparaben; PP – propylparaben.

Other abbreviations as in Table 1.

The relationship between phthalates and PCOS was investigated based on 5 published studies; 2 of them measured the concentration of phthalate metabolites in urine samples, another 2 in serum, and 1 publication demonstrated the level of phthalates in follicular fluid. The majority of the studies showed the positive association of phthalates with PCOS expressed as elevated cholesterol levels, insulin resistance indices, and blood triglycerides (Table 2). Just 1 study investigated the potential relationship of parabens with PCOS in plasma, demonstrating no association of parabens exposure with PCOS in women (Table 2). Also, only 1 study explored the potential association between UV filters, including BP-3, HMS, OC, in the urine samples, and PCOS. There was no significant relationship between PCOS and urine BP-3, nor is there an association between HMS concentration and PCOS. There was a positive association between OC and PCOS risk in obese and overweight women (Table 2).

This review included only 2 studies, 1 cross-sectional study and 1 case-control study on the relationship between TCS and PCOS. The first study discovered that infertile women with PCOS had significantly higher TCS urine levels than infertile women without PCOS (p = 0.0407). The second study found no association between urinary TCS and PCOS (Table 2). The cause of these results discrepancies may be linked with the study design, size of the population or the adjustment for potential confounding factors in the statistical analysis.

The studies included in review were well-prepared epidemiological studies mostly using case-control type of the study which in evaluation of such health effect seems to be proper. Only 6 studies used different study design (cross-sectional).

The health effect was based mostly on diagnosis of PCOS based on Rotterdam criteria, which are the most common diagnostic criteria for PCOS.

All of the presented studies used biological monitoring to assess the exposure which is the gold standard in such types of epidemiological studies. In most of the studies the non-persistent chemicals were assessed in urine [36, 38–40,43,56,57,86] whereas 8 studies were assessed in blood [33–35,37,42,44,45,58]. Only 1 limitation which can arise from the presented studies is using only 1 urine sample to confirm their exposure which in assessment of the non-persistent chemicals may not adequately measure the exposure. Human biomonitoring is a valuable tool to solve the issue. However, for non-persistent chemicals, it reflects the level of exposure only within a short time-frame prior to the collection of biological samples. At this point, the authors want to emphasize this limitation in interpreting exposure data. On the other hand according to previously published studies 1 urine sample is enough to confirm the exposure in epidemiological studies [87]. It is crucial to address some analytical aspects, particularly when analysing phthalates and bisphenols. The analysis of these compounds is complex due to potential contamination during sampling, storage, and processing, as well as the need for highly sensitive methods to detect low concentrations accurately. In this paper, the authors reference studies in which authors did not always employ analytical methods subject to rigorous quality control. For example, some studies lacked reported limits of detection (LOD) or quantification (LOQ), as well as internal or external laboratory quality control protocols [34,35,37,40,42,59]. As a result, the findings from such studies have methodological limitations and could impact the reliability and comparability of the reported data. Ensuring robust analytical practices is therefore essential to advancing research in this area. In most of the studies, potential confounding factors were used, such as, e.g., age, smoking status, body mass, study design, study population, biological fluids used for assessment. Most of the study population was recruited from gynecology and infertility centers, which guarantees a proper diagnosis of the recruited subjects.

The pathophysiology of the PCOS is unclear, and the influence of EDCs on PCOS is still not fully understood, but many countries have prohibited or limited to minimal concentration the use of non-persistent chemicals in everyday products.

Some studies presented in the review showed a positive association between BPA, phthalates, octocrylen, and PCOS. The data concerning TCS were inconclusive. There is no link between the exposures to parabens with PCOS. These studies highlight the urgent need for continued research on endocrine-disrupting chemicals (EDCs). The inconsistencies in the results may have been due to the different patient selection, different study population, different study protocols, and the collected sample of material being used for the study. This is why the main findings of the studies should be treated carefully. Moreover, given the widespread presence of EDCs in everyday products such as plastics, personal care items, and food packaging, it is important to understand their effects on human health. In addition, there is an increasing need for future research to investigate the causal relationships and underlying mechanisms between EDCs and PCOS.

In conclusion, this review publication suggests that there is an association between non-persistent endocrine disruptive chemicals and PCOS, especially for BPA, phthalates and OC. The results for paraben, BP-3, and HMS showed no correlation, and for TCS were inconclusive. However, further studies are essential to fully understand the impacts of non-persistent disrupting chemicals on PCOS. In summary, continued research and public awareness efforts for these important subjects are crucial for promoting safer alternatives and protecting human health.
